# Permutation Tests to Identify Significant Constraint or Promotion Within Biological Scatterplots

**DOI:** 10.1002/ece3.70584

**Published:** 2024-11-21

**Authors:** Anthony J. Mills, Ruan van Mazijk

**Affiliations:** ^1^ Department of Soil Science Stellenbosch University Stellenbosch South Africa; ^2^ C4 EcoSolutions (Pty) Ltd. Cape Town South Africa

**Keywords:** bivariate data, boundary line, non‐parametric statistics, quantile regression

## Abstract

Scatterplots of biological datasets often have no‐data zones, which suggest constraint or promotion of dependent variables. Although methods exist to estimate boundary lines—that is, to fit lines to the edges of scatters of data points—there are, to our knowledge, none available to assess the significance of the areal extents of no‐data zones. Accordingly, we propose a flexible boundary line definition paired with a permutation test of the magnitude of no‐data zones—rather than testing the shape or slope of the line as current methods do. Our proposed permutation test can be used with any method of defining a boundary line. We demonstrate our approach with empirical datasets, find no‐data zones that methods such as quantile regressions fail to detect, and discuss how our approach can quantify constraint and promotion relationships that are not always apparent with other statistics.

## Graphical abstract

Scatterplots of biological datasets often have no‐data zones, which suggest constraint or promotion of dependent variables by independent variables. We propose a flexible boundary line definition paired with a permutation test of the magnitude of areas without data—rather than testing the shape or slope of that line as previously developed methods do. Using empirical datasets, we demonstrate instances where—for example—quantile regressions fail to identify significant patterns.

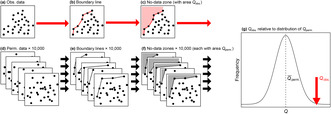

## 1 Introduction

**FIGURE 1:**
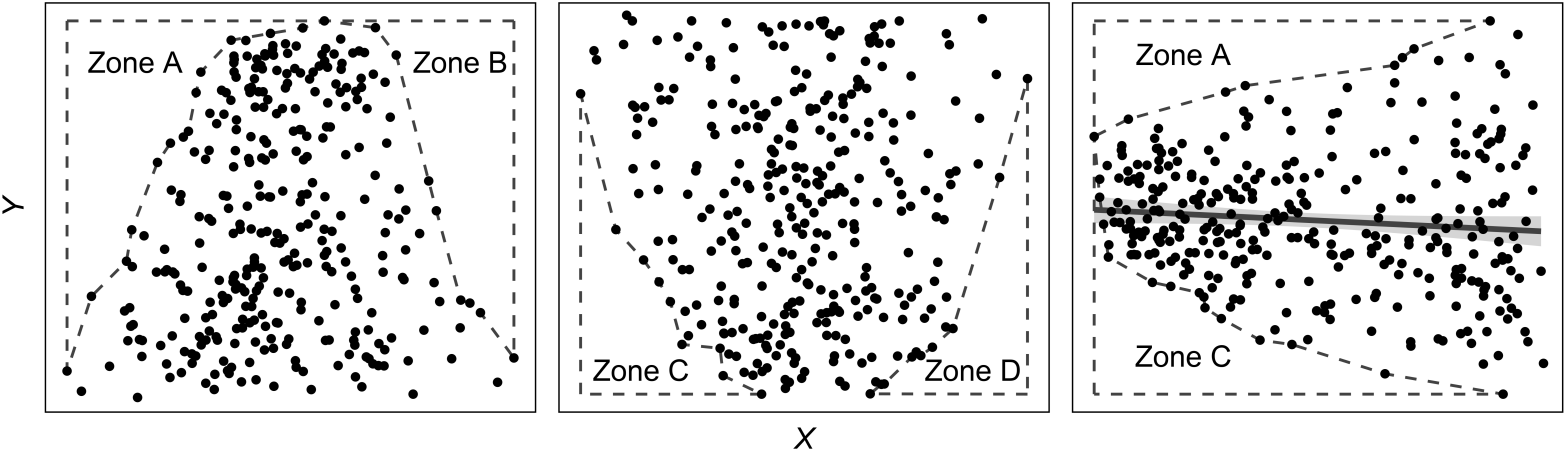
The four no‐data zones that can occur in scatterplots of biological data. Although such no‐data zones are often eye‐catching (Beitman 2009) and potentially of considerable conceptual significance (Guo, Brown, and Enquist 1998), biologists seldom investigate whether such patterns are statistically significant or merely illusory.

In biological datasets, one or more corners of a scatterplot are often free of data (Figure 1). Although lines can be drawn around the scatter of data points using methods such as mathematical models (Guo, Brown, and Enquist 1998; Hao et al. 2016; Li et al. 2022), partitioned regressions (Thomson et al. 1996), isolation of data points (Blackburn, Lawton, and Perry 2009), and quantile regression (Cade and Noon 2003; Cade, Terrell, and Schroeder 1999), there are to our knowledge no statistical methods available to calculate the probability that the area extents of these no‐data zones are greater than that expected by chance. A boundary line—also referred to as a constraint line, envelope (e.g., Hao et al. 2016) or limit line (Carling, Jonathan, and Su 2022)—delineates the edge of a scatter of data points as well as a zone where no data occurs. Values of the dependent variable are unlikely to occur beyond such an edge into the no‐data zone across a given range of the independent variable (Grubb 2016; Webb 1972). Since first mentioned (Webb 1972), boundary lines have been used throughout the biological sciences—particularly in ecology (e.g., Puglielli, Hutchings, and Laanisto 2021), agronomy (e.g., Evanylo and Sumner 1987; Schnug, Heym, and Achwan 1996; Walworth, Letzsch, and Sumner 1986), and forestry (e.g., Zhang et al. 2005). In all these disciplines, the probabilities of the sizes of no‐data zones are potentially of major biological significance (Guo, Brown, and Enquist 1998). This is because calculating them could assist in assessing whether the dependent variable is being constrained or promoted by the independent variable over a certain range of the independent variable (Milne, Ferguson, and Lark 2006; Walworth, Letzsch, and Sumner 1986; Webb 1972). Moreover, the presence of underlying boundary lines or triangular relationships (*sensu* Maller et al. 1983; Maller 1990) can be indicative of unmeasured factors—closely correlated with the independent variable—contributing to variation in the dependent variable (Grubb 2016; Hao et al. 2016; Mills et al. 2009).

In this paper, we describe: (i) a flexible and assumption‐free method to define a boundary line, delineating a no‐data zone; and (ii) a non‐parametric method to assess the significance of the position and magnitude of the no‐data zone—as opposed to assessing the shape or slope of the boundary line. Our method to assess the significance of no‐data zones is independent of the method used to define a boundary line.

## 2 Rationale and Related Methods

To illustrate the utility of an areal‐extent boundary line method and the features of a dataset it describes, we compare it with two other statistical methods often applied to bivariate scatterplot data: simple linear and quantile regression (Table 1). Our approach is not to present an exhaustive comparison of related methods but rather to illustrate the distinction between methods that assess the significance of the curves' parameters and methods that assess the significance of the area extent of no‐data zones. Quantile regressions—and other methods—simultaneously fit and test a line, whereas we propose a method to test the areas bound by a line. Although simple linear regression is inappropriate for defining a boundary line—as is evident below—we include its comparison as an introduction for readers unfamiliar with boundary line concepts and because it is so frequently used in ecology and broader biological sciences.

**TABLE 1:** Comparison of two statistical methods commonly applied to bivariate data (Cade and Noon 2003; Gotelli and Ellison 2004) and our proposed permutation test of no‐data zones. While the lines fit by quantile regressions can also be used to calculate the area extents of no‐data zones, the lines fit by linear regressions cannot.

	Linear regression	Quantile regression	Proposed permutation test of no‐data zones
Assumptions			
Independent observations	Yes	Yes	Yes
Residuals ~ *N*	Yes	No	Not applicable
Linear response	Yes	No	No
Homoscedasticity	Yes	No	No
Sensitivity to outliers	Some	Limited	Potentially extreme
Core estimate/statistic	Slope of mean	Slope of quantile	Area (*Q*) of no‐data zone
Null hypothesis (*H*_0_)	*H*_0_: slope = 0	*H*_0_: quantile slope = 0	*H*_0_: $Q={\bar{Q}}_{\text{perm}.}$
*p*‐value meaning	*P* (slope|*H*_0_)	*P* (quantile slope|*H*_0_)	*P* (*Q*|*H*_0_)
Appropriate as a boundary line method	No	Yes	Yes

Simple linear regression estimates the ‘line of best fit’—the mean value of the dependent variable conditional across the range of the independent variable, assuming the relationship follows a straight line. Although simple linear regression can be generalised to describe non‐linear relationships (e.g., with link functions), we only discuss linear forms of regression here to simplify comparison across methods. Quantile regression—an extension of linear regression (Koenker and Bassett 1978)—predicts a given quantile (*𝜏*) of the dependent variable conditional across the range of the independent variable. For example, if the model is specified to regress for the median (i.e., the 50% quantile; *𝜏* = 0.5), a quantile regression of the form *Y* = *β*_0_ + *β*_1_ *X*_1_ uses the median to estimate the central tendency of the dependent variable across the range of the independent variable. When specified with high or low quantiles (e.g., *𝜏* = 0.05 or 0.95), quantile regressions describe patterns in the extremes of the response variable, not the central tendency. Indeed, in many studies, this has been used to describe the ‘edges’ of the scatter of points to test whether the independent variable constrains or promotes the dependent variable (e.g., Anderson and Jetz 2005; Horning 2012; Kelt and Van Vuren 2001; Lessin, Dyer, and Goldberg 2001; Medinski et al. 2010; Mills et al. 2006, 2009, 2013; Strong 2011; Scharf, Juanes, and Sutherland 1998). However, constraint or promotion may be evident in features other than the shape of the extremes of a scatter of data points; it may also be evident in the ‘tightness’ of such relationships. For regression lines, the spread of data can be described with confidence intervals about the line or with metrics like *R* ^2^. Additionally, the shape of such spread can be described by regressing both an upper and lower quantile separately. For example, in datasets where the 95% quantile has a steep positive slope and the 5% quantile has a relatively shallow slope, the spread of the dependent variable increases as the independent variable increases—that is, a triangular relationship.

An underlying boundary line delineates the values of the dependent variable—across the range of the independent variable—beyond which observations are unlikely to occur. For example, the observed boundary lines for the upper‐left and ‐right no‐data zones (Figure 1) describe the maximum observed *Y*‐values for given observed *X*‐values. In our proposed method, the estimate of interest is the areal extent of regions of the scatterplot with no data—as opposed to, for example, the slope of the relationship between *X* and *Y* (Table 1). These regions are defined using the corners of the scatterplot, such that the areal extent of each no‐data zone is expressed relative to that of the full dataset. With the method we propose, the probability of a given no‐data zone of the observed extent is described relative to permutations of the raw data—the *p*‐value is associated with the area (*Q*; Table 1) of the no‐data zone. This contrasts with the *P*‐values associated with the slope terms of linear and quantile regressions—that is, the probability that the observed trend in the dependent variable is a product of chance. In those cases, the *p*‐values are associated with the locations and slopes of relationships between *X* and *Y* (Table 1). Although confidence intervals about such regression lines can also be used to describe the likely range of values for the dependent variable, these intervals are based on the statistic that a regression estimates (i.e., the slope) and not an estimate of where data is likely—or unlikely—to occur. We postulate that investigating constraint or promotion effects is better done by assessing the magnitude by which the dependent variable is restricted rather than assessing the shape (i.e., slope) of that restriction.

In Figure 1, for example, it would be useful to know whether no‐data Zone A is greater in area extent than would be expected by chance. If it is greater, then it would be reasonable to conclude that the dependent variable is constrained by the independent variable—or a closely correlated variable—over the first third of the range of the independent variable. Similarly, if Zone D is greater than expected by chance, then the dependent variable is likely to be promoted by the independent variable—or a closely correlated variable—over the last third of the range of the independent variable.

## 3 Defining a Boundary Line

To test the significance of the area extent of a no‐data zone, a first step is delineating the edge of that no‐data zone—that is, identifying which points in the scatterplot form the ‘boundary’. Such lines can be defined with multiple approaches—for example, quantile regressions (Mills et al. 2006; Grubb 2016), Pareto fronts (Shoval et al. 2012; Sheftel et al. 2013), convex hulls, or kernel density estimation (Carmona, Pavanetto, and Puglielli 2024). The most flexible and assumption‐free definition, we propose, uses the four extreme points of the scatter—the data points with the minimum and maximum *X*‐ and *Y*‐values. To delineate such a boundary, straight lines are used to join points—progressively nearer the centre of the scatter in one direction but further from the centre in the direction perpendicular to that—starting from one of the four extreme points. The resulting boundary line is equivalent to a Pareto front. Using the boundary line for Zone A as an example, the procedure is described below.

1. Start with the point with the minimum *X*‐value.
2. Only considering points with *X*‐ and *Y*‐values greater than those of the starting point, select the point closest in the *X*‐direction to connect to the boundary.
3. Repeat steps 1 and 2, with the selected point as the new starting point, until there are no points with greater *Y*‐values remaining.
4. Connect the resulting sequence of points with the corner of the plot—that is, an imaginary point at the horizontal minimum and vertical maximum coordinates of the scatterplot.

**FIGURE 2:**
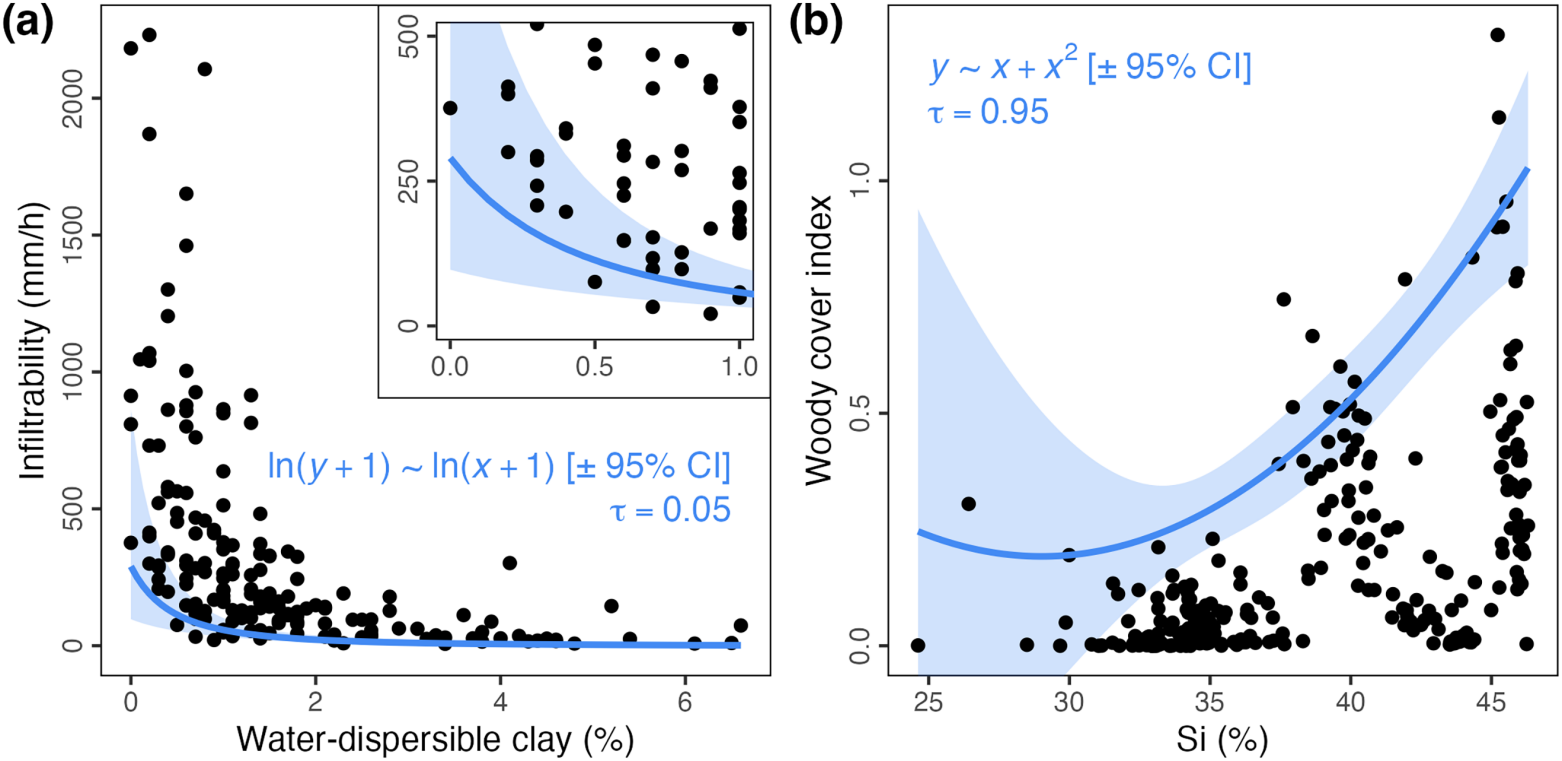
Application of quantile regressions to empirical datasets, for comparison with our proposed boundary line method (as in Figure 3a,c). The forms (log–log and parabolic, respectively) and quantiles (*𝜏*; 5% and 95%, respectively) of each relationship regressed are noted in blue. Pale‐blue bands represent the 95% confidence intervals (CI) about the regression lines. In (a), the inset highlights the lower‐left corner of the data (as in Figure 3a), with the same quantile regression plotted.

The area of this polygon can then be computed. As boundary lines can be defined in other ways, we suggest that in such cases the curve described be used to delineate the no‐data zone along with the corners of the scatterplot. The significances of the areal extents of no‐data zones can then be tested with the permutation method we describe here. Using regression methods to delineate boundary lines—irrespective of the significance of the slope of that regression—has its limitations, in that the form of the relationship must be defined a priori and is forced to be a smooth line. This can result in seemingly inappropriate or uncertain fits (e.g., Figures 2b versus 3c). Kernel density estimation methods, while more flexible than regression lines, are dependent on a priori or parametric bandwidth selection (e.g., Steury et al. 2010). It is, however, noteworthy that both regression and kernel density estimation methods are less statistically sensitive to outliers than the boundary line procedure described above (Table 1).

**FIGURE 3:**
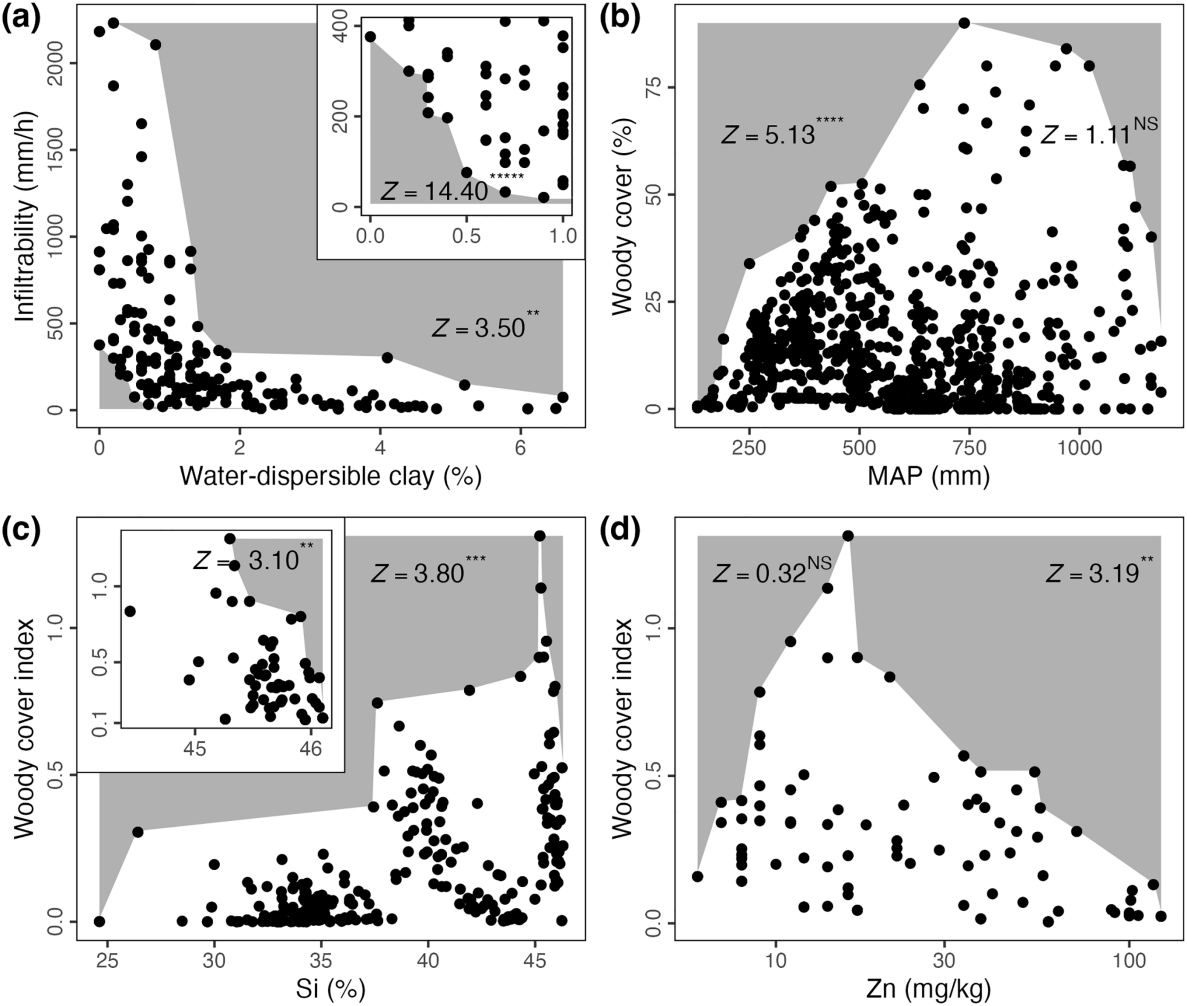
Application of our proposed boundary line permutation test to empirical datasets. (a) Infiltrability versus water‐dispersible clay content of soils across Namibia and western South Africa (Mills et al. 2006), with the lower‐left no‐data zone highlighted in the inset. (b) Woody cover versus mean annual precipitation (MAP) in African woody savannas (Sankaran et al. 2005), (c, d) Woody cover index versus (c) silicon (Si) and (d) zinc (Zn) content of soils across Namibia and western South Africa (Mills et al. 2013). In (c), the upper‐right no‐data zone highlighted in the inset depicts only points from true woodland savanna sites (i.e., excluding desert, Succulent Karoo, Nama Karoo and thornbush savanna sites); the *Z*‐value for this no‐data zone results from a permutation test of only those data. All tests were based on 10,000 permutations of their respective data. Asterisks denote significances (*p* ≤ 1 × 10^−10^, *****; *p* ≤ 1 × 10^−6^, ****; *p* ≤ 1 × 10^−4^, ***; *p* ≤ 0.001, **; *p* ≤ 0.005, *; *p* > 0.05, not significant—NS) following one‐sided *Z*‐tests of each observed boundary line against 10,000 permutations.

## 4 Permutation Testing

As is common practice in ecology and evolutionary biology, we apply a non‐parametric test (Gotelli and Ellison 2004; Ives 2022; Pielou 1966) to our measure of interest—that is, the areal extent of a no‐data zone. In instances where parametric null distributions and observed no‐data zones need to be compared for a priori reasons, permutations can be generated from Monte Carlo simulations. For example, Díaz et al. (2016) and Puglielli, Hutchings, and Laanisto (2021) tested the significances of the observed magnitudes (in those cases, volumes) of data in trait space using Monte Carlo approaches. While those studies did not test the significances of no‐data zones, Monte Carlo simulations could be used to parametrically generate a null distribution of such zones.

**FIGURE 4:**
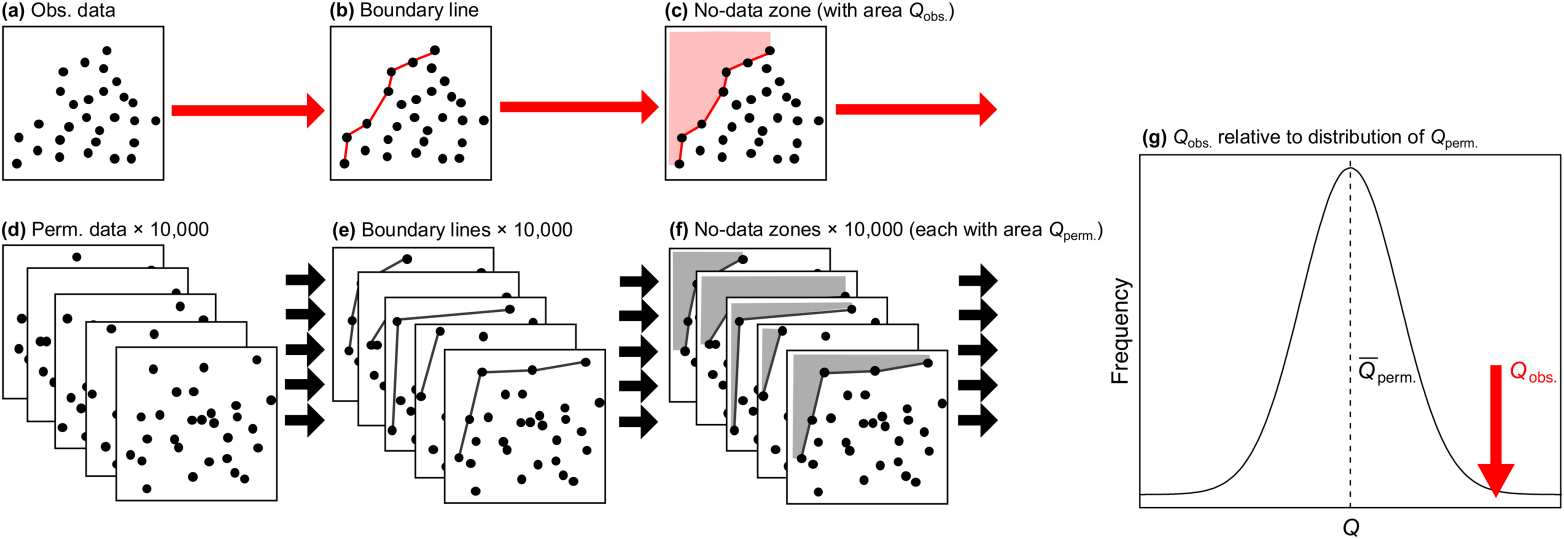
The logic of the permutation test for a boundary line for the upper‐right no‐data zone, illustrating how the areal extents of the (a–c) observed no‐data zone (*Q*_obs._) and (d–f) 10,000 permuted no‐data zones (each *Q*_perm._) are compared in (g) a *Z*‐test.

**FIGURE 5:**
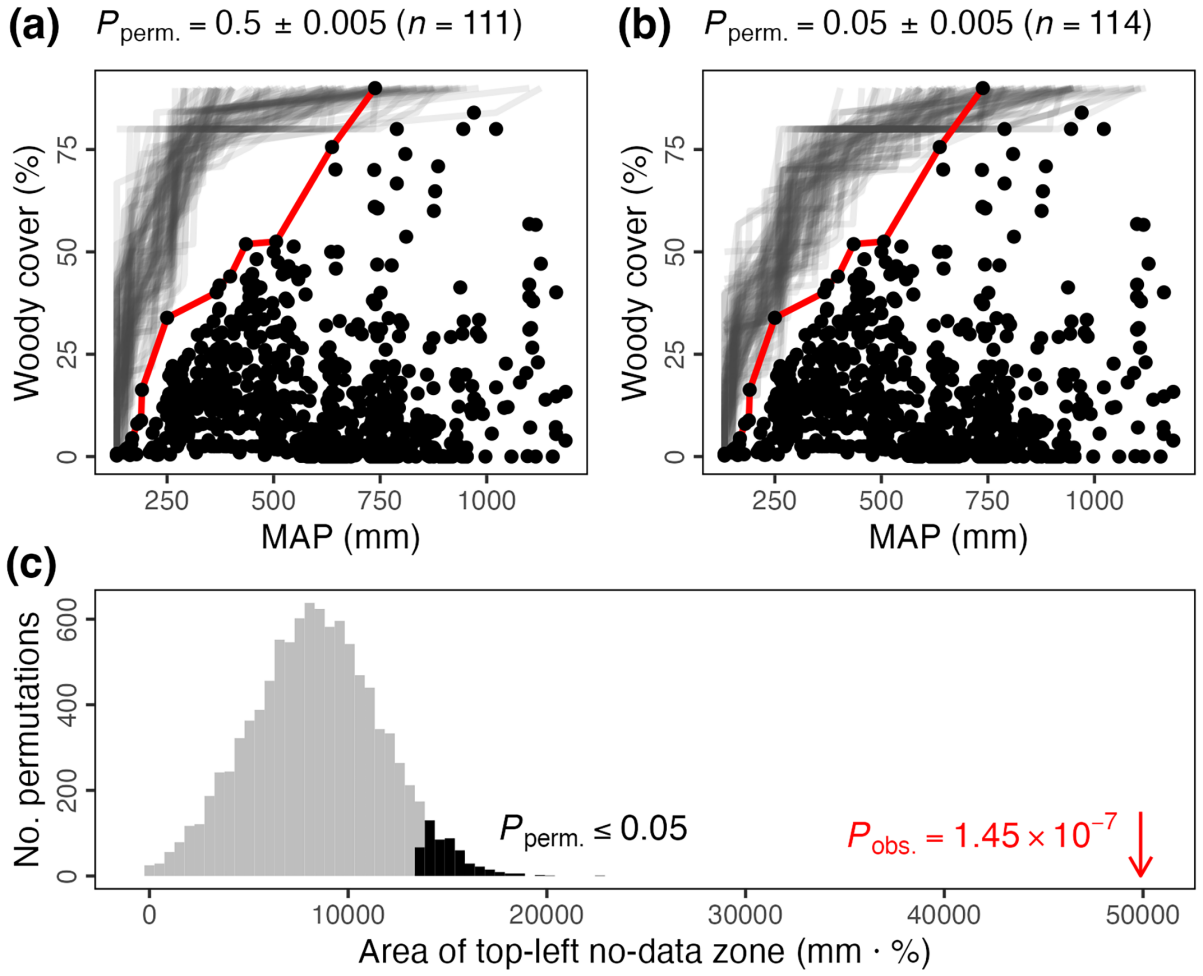
The logic of the permutation test for a boundary line using empirical data (as in Figure 3b; Sankaran et al. 2005), testing the upper‐left no‐data zone. Boundary lines were defined for each of the 10,000 permutations of the data (as in Figure 4d–f). Relative to these, the significance of the area bounded by the observed boundary line (in red, as in Figure 4a–c) is assessed using a one‐sided *Z*‐test (c). Each permutation's boundary line can also be subjected to a *Z*‐test relative to the other 9999 lines. Subsets of these permuted boundary lines are presented in translucent grey in (a) and (b) (numbering 111 and 114, respectively) according to their *p*‐values: (a) those close to 0.5 (i.e., examples that are not significant) and (b) close to 0.05 (visualising where significant lines typically fall), respectively.

Here, we propose a permutation‐based method for assessing the significance of an observed boundary line's corresponding no‐data zone (Figure 4)—irrespective of how the boundary was defined. The proposed method uses permutations of the observed data to generate a random null distribution against which the observed data is compared. Using Zone A as an example again (see Figure 5, illustrated with empirical data), the procedure is described below.

1. Measure the area of Zone A in the observed data.
2. Randomly reorder the *X* and *Y* coordinates separately, creating a new dataset of *X–Y* data point pairs (but comprising the same *X*‐ and *Y‐*values).
3. Measure the area of Zone A in the new permuted dataset.
4. Repeat steps 2 and 3 a total of 10,000 times, for example.
5. Calculate the average extent of the 10,000 Zone A areas generated in step 4.
6. Use a one‐tailed *Z*‐test (see Figure 5c) to determine the probability of the area calculated in step 1 being greater than the average area calculated in step 5.

Described mathematically, the vectors **X** and **Y** represent the coordinates for a series of observations (*x*_*i*_ and *y*_*i*_), such that the area *Q*_obs._ of a given no‐data zone (A, B, C or D) is based on some function of **X**_obs._ and **Y**_obs._, as described above (see also Figure 4a–c). Sampling without replacement, one can permute **X**_obs._ and **Y**_obs._, giving **X**_perm._ and **Y**_perm._ (Figure 4d), and determine a given no‐data zone's area (*Q*_perm._) accordingly (Figure 4f). Doing this, for example, 10,000 times generates a set of 10,000 areas based on random permutations of the data, such that *Q*_perm._ follows a normal distribution (Figure 4g). The probability of observing an area at least as great as a given *Q*_obs._ can then be calculated using a one‐tailed *Z*‐test—that is, the difference between *Q*_obs._ and the mean of *Q*_perm._ (${\bar{Q}}_{\text{perm}.}$), in terms of the standard deviation of *Q*_perm._ (*s*):

$$Z=\frac{Q_{\mathrm{obs}.}-{\bar{Q}}_{\text{perm}.}}{s}$$

## 5 Examples With Empirical Data

In addition to the empirical data used to illustrate the logic of our proposed permutation test (Figure 5) (Sankaran et al. 2005), we also demonstrate here further example applications of this test to other empirical datasets (Figure 3) (Mills et al. 2006, 2013).

When comparing soil infiltrability and water‐dispersible clay content (Figure 3a), permutation tests identify significant no‐data Zones B and C—that is, a significant lack of observations where infiltrability and water‐dispersible clay content are great (Zone B) as well as small (Zone C). While similar conclusions can be drawn using quantile regression (e.g., for Zone C; Figure 2a), the latter method estimates a smaller no‐data zone than our proposed method, with relatively wide confidence intervals.

The extents to which woody cover is constrained at different levels of rainfall, soil silicon and soil zinc (Figure 3b–d) all exemplify the case of ‘humped’ constraint–promotion datasets. Our method distinguishes between patterns of constraint in these datasets that are significant (minor amounts of rainfall and soil silicon, and large amounts of soil zinc) and illusory (large amounts of rainfall and soil silicon, and minor amounts of soil zinc) (Figure 3b–d). To test for a humped relationship with another method, we performed a quantile regression with a parabolic form between woody cover and soil silicon. This quantile regression (Figure 2b) failed to identify and highlight the significance of no‐data Zone B (as in Figure 3c). Furthermore, the non‐parametric observed boundary line in Figure 3c shows the easily undetected—yet ecologically important—pattern of decreasing woody cover as soil silicon increases beyond 45%. This contrasts with the easily detected pattern shown with the parabolic quantile regression in Figure 2b of increasing woody cover as soil silicon increases above 30%. A shortcoming of regression methods is apparent here: the form of the relationship to be fit must be explicit and chosen a priori. The inappropriate fit (Figure 2b) is specific to parabolic curves: parabolas are necessarily symmetrical, while the humped pattern of woody cover versus silicon is potentially asymmetrical. As permutation tests of observed boundary lines can be performed regardless of how they are defined, our method offers the flexibility to accommodate such cases. For example, if kernel density estimates are used to delineate the boundary line, the significance of the no‐data zone bounded by that line can also be assessed using our method.

In addition to the above demonstrations of the significance of observed no‐data zones, we note that this significance is sensitive to the spread of the rest of the dataset used in permutation tests. When applying our method to the full woody cover versus soil silicon dataset, Zone B is not significant (Figure 3c). However, Zone B is significant when only considering the woodland‐savanna subset of that data (inset in Figure 3c). Notwithstanding the value of determining the significances of no‐data zones in subsets of data, we encourage the use of our method with prudence, applying it only to subsets of data that have been predetermined in the specific context of an investigation.

## 6 Concluding Remarks

Our proposed permutation test of observed boundary lines could be applied to a variety of datasets, not just ecological or agronomic—for example, soil temperature dynamics (Chmura et al. 2023), physical properties of cancer cells (Naghavian et al. 2023), neurons in the visual cortex (Fişek et al. 2023), cloud cover dynamics (Vo et al. 2023), and planetary temperatures (Peterson et al. 2023). Across datasets as varied as these, this method is likely to assist in describing relationships that are not immediately apparent with regression lines.

## Author Contributions

**Anthony J. Mills:** conceptualization (lead), formal analysis (equal), methodology (lead), visualization (supporting), writing – original draft (lead), writing – review and editing (equal). **Ruan van Mazijk:** data curation (lead), formal analysis (equal), methodology (supporting), software (lead), visualization (lead), writing – original draft (supporting), writing – review and editing (equal).

## Conflicts of Interest

The authors declare no conflicts of interest.

## Data Availability

The authors have nothing to report.
